# Field-Isolated Genotypes of *Mycobacterium bovis* Vary in Virulence and Influence Case Pathology but Do Not Affect Outbreak Size

**DOI:** 10.1371/journal.pone.0074503

**Published:** 2013-09-23

**Authors:** David M. Wright, Adrian R. Allen, Thomas R. Mallon, Stanley W. J. McDowell, Stephen C. Bishop, Elizabeth J. Glass, Mairead L. Bermingham, John A. Woolliams, Robin A. Skuce

**Affiliations:** 1 School of Biological Sciences, Queen’s University Belfast, Belfast, Northern Ireland, United Kingdom; 2 Veterinary Sciences Division, Bacteriology Branch, Agri-Food and Biosciences Institute, Belfast, Northern Ireland, United Kingdom; 3 The Roslin Institute and Royal (Dick) School of Veterinary Studies, University of Edinburgh, Midlothian, Scotland, United Kingdom; University College Dublin, Ireland

## Abstract

Strains of many infectious agents differ in fundamental epidemiological parameters including transmissibility, virulence and pathology. We investigated whether genotypes of *Mycobacterium bovis* (the causative agent of bovine tuberculosis, bTB) differ significantly in transmissibility and virulence, combining data from a nine-year survey of the genetic structure of the *M. bovis* population in Northern Ireland with detailed records of the cattle population during the same period. We used the size of herd breakdowns as a proxy measure of transmissibility and the proportion of skin test positive animals (reactors) that were visibly lesioned as a measure of virulence. Average breakdown size increased with herd size and varied depending on the manner of detection (routine herd testing or tracing of infectious contacts) but we found no significant variation among *M. bovis* genotypes in breakdown size once these factors had been accounted for. However breakdowns due to some genotypes had a greater proportion of lesioned reactors than others, indicating that there may be variation in virulence among genotypes. These findings indicate that the current bTB control programme may be detecting infected herds sufficiently quickly so that differences in virulence are not manifested in terms of outbreak sizes. We also investigated whether pathology of infected cattle varied according to *M. bovis* genotype, analysing the distribution of lesions recorded at post mortem inspection. We concentrated on the proportion of cases lesioned in the lower respiratory tract, which can indicate the relative importance of the respiratory and alimentary routes of infection. The distribution of lesions varied among genotypes and with cattle age and there were also subtle differences among breeds. Age and breed differences may be related to differences in susceptibility and husbandry, but reasons for variation in lesion distribution among genotypes require further investigation.

## Introduction

Bacterial pathogens are frequently classified into distinct strains according to virulence, detectability, host specificity and other parameters that determine the magnitude of their impact on host populations; classifications which may then be used to assist and improve disease management. For example, laboratory trials have found evidence of variation in virulence among clinical strains of *Mycobacterium tuberculosis,* the causative agent of human tuberculosis [1]–[3]. As genotyping technologies have advanced, classification of strains according to genetic similarity has become more common [4], often followed by efforts to detect phenotypic variation among strains that were originally distinguished using molecular techniques. Variation in immunogenicity, virulence and pathology has been found among the six major lineages of *M. tuberculosis*, along with evidence of host-pathogen coevolution in regions where lineages are long established [5]–[7].

We investigated whether genotypically-distinct strains of *M. bovis* differ in transmissibility and virulence, and whether an aspect of the pathology of infected cattle varies according to pathogen genotype. Bovine tuberculosis is a chronic disease of farmed cattle and wildlife which may also be transmitted to humans, presenting a public health risk [8], [9]. In the UK, a system of regular skin testing followed by compulsory slaughter of infected animals, supported by active abattoir surveillance, is used in an attempt to control bTB incidence in the cattle population [10]. This programme imposes significant costs on the UK cattle industry and government. In England alone, the bTB control programme costs an estimated £91 million annually, comprised mostly of testing costs and compensation for farmers [11].

Since the late 1990s a large number of *M. bovis* isolates from infected cattle in the UK have been genotyped to help trace sources of infection [12], [13]. This provides a unique opportunity to assess whether there is phenotypic variation among genotypes and whether knowledge of such variation might be exploited to aid control of the epidemic. A similar approach has been used to compare strains of *M. tuberculosis* infecting human populations, with some strains more likely to be found in clusters of cases, indicating greater virulence or transmissibility [14], [15].

In the UK, cattle herds in which *M. bovis* is detected are placed under movement restrictions until all infected animals have been removed (a herd breakdown), and so the national bTB epidemic consists of a series of discrete breakdown events that vary in the number of animals infected. A survey of bTB outbreaks within herds in Great Britain revealed subtle differences among pathogen genotypes in outbreak size and the proportion of cases visibly lesioned [16], leading to speculation that closely related *M. bovis* genotypes might vary in transmissibility. We investigated these effects, conducting a larger scale analysis whilst accounting for variation in the host population structure (especially herd size and mix of cattle breeds) within Northern Ireland, where the *M. bovis* population has been systematically sampled.

Strains of *M. tuberculosis* have also been shown to induce distinctive pathologies in humans, with some lineages associated with a greater proportion of extra-pulmonary cases that carry an increased risk of mortality [6], but no previous studies have searched for *M. bovis* genotype-specific variation in cattle pathology. The site of the initial infection can be deduced from the location of tuberculous lesions, provided that infection has not progressed to multiple sites, and is thought to be indicative of the route of infection [17], [18]. In naturally infected cattle in the UK, lesions are most commonly found in lymph nodes draining the respiratory tract, indicating that inhalation is the primary route of infection [19]. However, lesion distribution and severity may be modified by cattle breed and husbandry; Holstein cattle in Ethiopia allowed to graze extensively were shown to have a greater proportion of lesions in the upper respiratory tract and mesenteric lymph nodes than animals kept indoors under intensive conditions, a pattern indicative of infection via ingestion [20]. In the UK, dairy animals are typically managed more intensively than beef animals so we might expect to find variation in lesion distribution among the major beef and dairy breeds.

In this study we used a large population wide survey of *M. bovis* genotypes in Northern Ireland [13] linked with cattle population records, to assess whether there is phenotypic variation among *M. bovis* genotypes. We estimated the relative transmissibility of *M. bovis* genotypes by analysing the distribution of outbreak sizes. We also investigated whether there is variation among *M. bovis* genotypes in virulence, measuring the proportion of those cattle in each breakdown that tested positive (using the single intradermal comparative tuberculin test, henceforth skin test) that were subsequently found to have tuberculous lesions. Finally, we investigated bTB pathology, specifically the influence of *M. bovis* genotype and cattle breed and age on the proportion of infected animals that were lesioned and the distribution of lesions in these animals.

## Methods

### Outbreak Detection and Genotyping

The bTB control programme in Northern Ireland is based on a regime of annual skin testing of all animals and post-mortem inspection for tuberculous lesions [10], [21]. Following detection of bTB by either method, the infected herd is placed under restrictions whereby cattle can only be moved if they are sent directly to slaughter, with skin test positive animals (reactors) being dispatched immediately. All animals in the herd are then subjected to repeated skin tests at sixty day intervals and tissue samples from reactors and lesioned animals are subjected to histopathological tests and laboratory culture to confirm infection with *M. bovis*. If infection is confirmed, all remaining animals in the herd must undergo two successive negative skin tests before restrictions are lifted and a breakdown is deemed over. If infection is not confirmed then a single clear herd test is sufficient for the breakdown to be ended. In 2011 there were approximately 1.6 million cattle in Northern Ireland in ca. 25,000 herds, with the herd incidence of bTB close to 5%.

We combined data from a nine year (2003–2011) survey of the genetic structure of the *M. bovis* population with detailed records of the cattle population during the same period. Beginning in 2003, a single isolate has been genotyped from each newly confirmed herd breakdown, provided that there had been no confirmed cases in the herd during the previous 365 days. Sampling increased to two isolates per breakdown in 2006 and to every confirmed isolate in June 2009. Genotypes were defined using a combination of spoligotyping and VNTR (variable number of tandem repeat) markers selected to provide maximum resolution of the clonal relationships among herd breakdowns (VNTR markers discriminate within spoligotypes) [22]. A total of 23,711 isolates were genotyped during the study period, covering 11,818 herd breakdowns with at least one isolate genotyped. The majority of the 351 genotypes identified were rare (number of breakdowns for each genotype: median = 2, range 1–4443; 289 genotypes were found in less than 10 breakdowns each), and there was pronounced inter-annual variation in relative frequency of occurrence [13]. We extracted corresponding records detailing skin tests, animal life histories and movements among herds from the Animal and Public Health Information System [23], a database administered by the Department of Agriculture and Rural Development.

### Breakdown Size

We estimated the average size of breakdowns caused by *M. bovis* genotypes, defining breakdown size as the total number of animals detected with bTB during the period of movement restriction. There were 1892 herd breakdowns between June 2009 and December 2011 in which all lesioned cases were genotyped (5066 isolates). From these we excluded 207 breakdowns in which multiple genotypes were detected because in instances where reactors were not visibly lesioned it would not have been possible to identify the relative contribution of each genotype to the breakdown size. The mean length of herd breakdowns was seven months but a small number of herds remained under restrictions for much longer. Many of these were beef finishing herds that buy in large numbers of cattle from many different sources and which sell directly to abattoirs. Persistent breakdowns in these herds are likely to be the result of multiple imported infections and we therefore excluded 61 outbreaks that lasted longer than fourteen months (the upper 90% quantile of all breakdown durations over the period 1993 to 2012). Following these exclusions 1624 breakdowns remained (89% of fully genotyped breakdowns) with 87 different genotypes represented.

We fitted a series of models to examine the relative influences of pathogen genotype, herd size and the means by which infection was detected on breakdown size. In the UK and Ireland larger herd sizes have been associated with both increased risk of herd breakdown [24], [25] and persistent infection within herds [26], [27]. In Northern Ireland cattle are housed over winter in large sheds with shared airspace. In addition, although housed cattle are often batched according to age and sex there is likely to be occasional physical contact among batches when animals are moved for veterinary treatment and other routine management. Large herds are typically housed in larger sheds designed to enable mechanized feeding, rather than in an increased number of small units (pers. obs.). Therefore an infected animal in a large herd may have contact with a larger number of susceptible animals and so we expected breakdown size to increase with herd size. The majority of breakdowns were detected either as a result of annual testing and abattoir surveillance but 31% resulted from epidemiological investigations into other breakdowns (tracing of infectious contacts). We expected these breakdowns to differ in size in comparison with routine detections because the different tracing methods give an indication of the probability and timescale of disease presence in the herd (Table 1).

**Table 1:** Modes of detection for bTB breakdowns in Northern Ireland and predicted effects on breakdown size.

Mode of detection	Predicted effect on breakdown size	Reason
AHT – annual herd test	baseline	
LRS – lesions found at routine slaughter	−	Slaughter usually more frequent than AHT therefore less time for infection to spread.
LCT – lateral check test (herds sharing a boundary with infected herd are tested)	−	Less time for infection to spread since last herd test than under annual testing.
BCT – backward check test (source herds of cattle bought into focal herd are tested)	+	Onward spread to another herd has occurred so likely to be a large number infected in the source herd.
CTS – check test status (individual animals withID or movement queries tested)	+	Animals bought in with unknown disease status potentially increase risk to the remainder of the herd
FCT – forward check test (destination herds of animals that left focal herd immediately prior to a breakdown)	−	Short period since potentially infectious animal(s) moved in to herd and so little time for large outbreak to develop.
CTT – check test trace (forward trace of individual animal)	−	As above.

We tested whether herd size or contact tracing had a significant influence on breakdown size by comparing the fit of generalized linear mixed models (GLMMs) incorporating different combinations of these factors as fixed effects. We fitted four models of increasing complexity; M1) no fixed effects (null model) M2) just herd size, M3) just contact tracing and M4) both herd size and contact tracing. In our dataset there were seven different situations by which breakdowns were detected (Table 1) and we estimated a coefficient representing each in models incorporating contact tracing (i.e. varying the intercept). A single additional coefficient (the regression slope) was estimated in models including herd size, representing a linear relationship between the logarithm of herd size and breakdown size (this functional form provided the best fitting models among various approaches tested: ordinary linear models, polynomial fits and treating herd size as a categorical variable). Herd sizes fluctuate throughout the year, peaking in summer months. Therefore, we used total number of animals that had been present in the herd over the previous calendar year as our measure of herd size. In all models genotype effects were included as normally distributed random variables. This mixed modelling approach allowed us to account for uncertainty around estimates for genotypes that were responsible for very few outbreaks; estimates for these are regressed towards the overall mean and have wider confidence intervals. We used a Poisson error distribution with a log link, but noted that the data were overdispersed, with more large outbreaks than expected based on a Poisson model. Following the approach of Elston et al. [28] we explicitly modelled this extra variance by including outbreak effects as normally distributed random variables, nested within genotype effects (i.e. fitting a data level random variable) resulting in a Poisson-lognormal model where each outbreak is associated with variation at both the outbreak and higher hierarchical levels. Models were fitted using the *lme4* package [29] in *R 13.2* [30]. We also searched for variation among genotypes at the coarser (spoligotype) level of discrimination, fitting a similar series of models with effects of VNTR types nested within spoligotypes.

We compared the candidate models using Akaike’s Information Criterion (marginal) which scores models according to their complexity and fit to the data (models are penalised for each parameter estimated). In a given set, models with lower AIC are considered to be better supported by the data and those with AIC values that differ by more than two are considered to be significantly different [31]. We then examined the estimated parameters from the best fitting model to see if they supported our predictions about outbreak size and contact tracing (Table 1). Using a likelihood ratio test we also compared the best fitting model with a simpler model with the same fixed effect structure but with no genotype effects.

### Virulence

**Figure 1:**
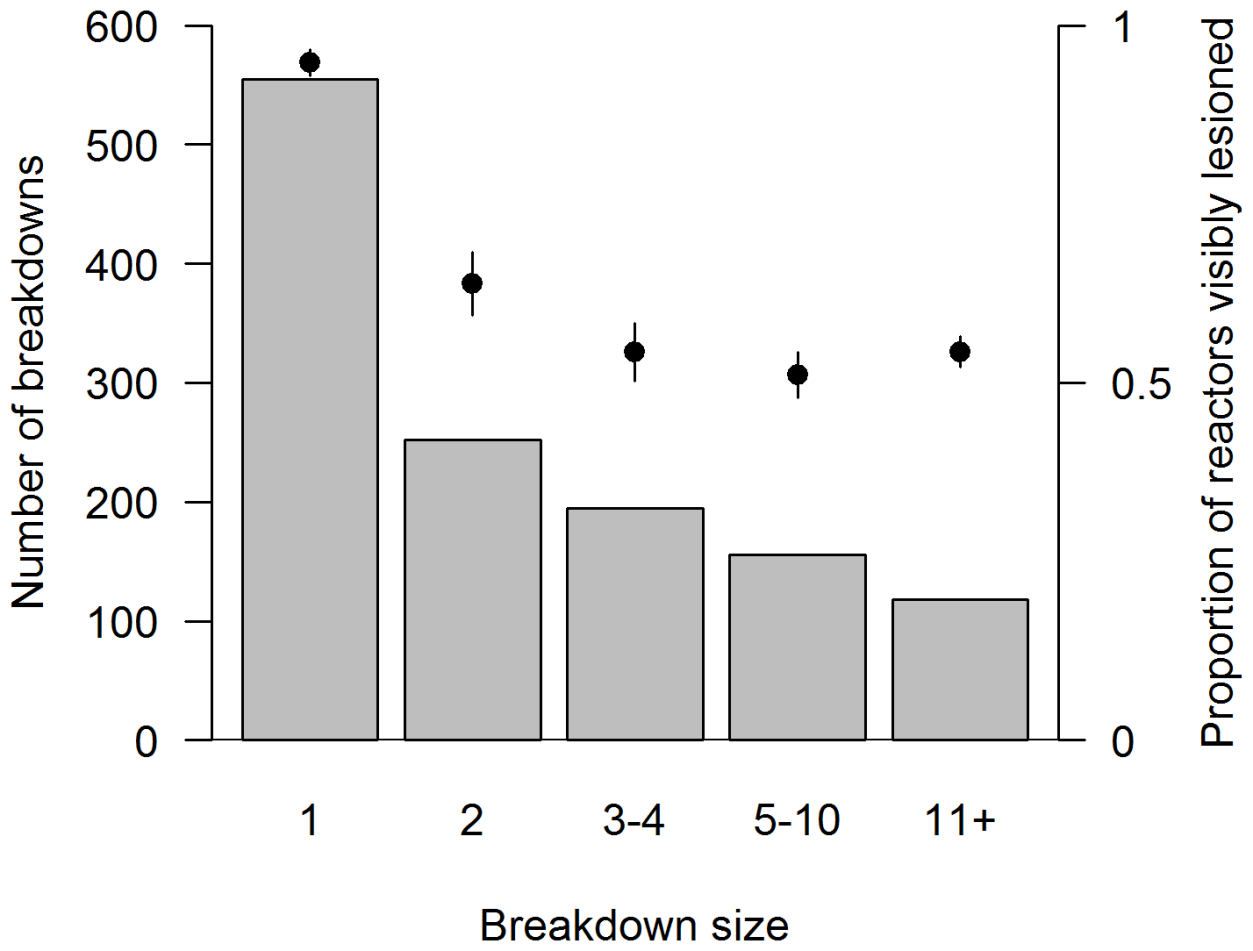
Herd breakdown sizes and proportion of reactors visibly lesioned in Northern Ireland 2009–2011. Distribution of herd breakdown sizes (grey bars). Points and error bars indicate average proportion of reactors found to be visibly lesioned in breakdowns of each size class (mean and 95% CIs).

We estimated the proportion of reactors that were found to have tuberculous lesions as a measure of genotype virulence, whilst attempting to control for variation in disease susceptibility among cattle of different ages and breeds. Of the records used for the breakdown size analysis, we selected only those that had at least one reactor (in some breakdowns all cases were detected at abattoir), a subset of 1276 breakdowns with a total of 4706 post mortem records of reactors, 59% of which were lesioned. We modelled the mean proportion of reactors that were lesioned using a logistic GLMM with genotype and breed effects incorporated as normally distributed random variables and with animal age (in months) as a fixed effect. Exploratory analysis showed that the proportion of reactors lesioned depended on the breakdown size. Reactors in breakdowns with one or two reactors were more likely to be lesioned than those in larger outbreaks (Figure 1). This discrepancy is probably related to the ways in which the skin test is interpreted in different sized breakdowns. If there are a large number of reactors detected in a herd, a more severe interpretation of the skin test may be applied to try to ‘clean’ the herd of infected animals. Animals with inconclusive responses to the skin test might be culled, and these may be in very early stages of infection and so be unlikely to have gross lesions. To ensure that this factor did not bias our estimates of genotype virulence we fitted the model twice, first using records from all breakdowns and secondly using only breakdowns with more than two reactors. We then correlated the estimated genotype effects from the two models. We also tested whether the proportion of reactors lesioned varied depending on the manner of disease detection because contact tracing may often disclose outbreaks at an earlier stage than annual skin testing, and hence cases may be less advanced (Table 1). We fitted a second model estimating additional parameters for each form of tracing (i.e. fitting these as fixed effects) but retaining the random genotype effects. The two models were then compared by means of a likelihood ratio test.

### Lesion Distribution

We aimed to determine whether the presumed primary site of infection varied depending on *M. bovis* genotype or host age and breed. The distribution of lesions in both experimentally- and naturally-infected animals is considered to be indicative of the primary site of infection, with the majority of lesions found in lymph nodes draining the respiratory tract [17]–[19]. We selected post mortem records of all reactors from which *M. bovis* had been successfully isolated and genotyped between 2003 and 2011, a total of 16,571 animals. We did not consider animals that were found to be lesioned at routine slaughter because abattoirs vary considerably in the quality of inspection for lesions [32], [33]. However, a single abattoir handled 85% of reactors in our sample, minimising bias caused by different inspection regimes. Animals with multiple lesions (approximately 20% of reactors) were also excluded because in these cases it would not have been possible to determine which lesion was closest to the initial site of infection. We also excluded cases where lesions were likely to have resulted from haematogenous spread rather than being close to the site of initial infection (e.g. lesions in the popliteal or prescapular lymph nodes or in the liver). Following these exclusions, a total of 12,633 post mortem records remained. Cases were classified into three groups based on the site at which lesions were found; infection of the upper respiratory tract was indicated by lesions in the head lymph nodes, the lower respiratory tract was represented by lesions in the lungs or the bronchio-mediastinal lymph nodes and the digestive tract was represented by lesions in the intestines or mesenteric lymph nodes.

We modelled the proportion of respiratory tract lesions that were found in the lower section of the tract using a logistic GLMM to determine whether there were differences in lesion distribution among genotypes and cattle of different ages and breeds. Genotype and breed effects were incorporated as normally distributed random variables and animal age (in months) was incorporated as a fixed effect. Finally we correlated the estimated genotype effects from this model with those estimated for virulence.

## Results

### Breakdown Size

Breakdown size was influenced by both herd size and contact tracing but not significantly by pathogen genotype. Breakdowns ranged in size from one to 73 infected animals but the majority were small with 75% having three cases or fewer. Model M4, incorporating herd size and contact tracing was best supported by the data and was significantly better than the other candidate models (ΔAIC >10 in each case, Table 2). The estimated mean size of breakdowns (number of infected animals) detected at annual herd tests was 2.00 (95% CI: 1.90–2.11) and predicted breakdown size increased significantly with the logarithm of herd size (Table 3). Breakdowns detected by backward or lateral tracing of infected animals were significantly larger than those detected by annual herd tests. There was no significant difference in the mean sizes of breakdowns detected by annual herd tests and forward tracing. Breakdowns detected as a result of abattoir surveillance were significantly smaller than those detected by annual herd tests (Table 2).

**Table 2:** Comparison of candidate models explaining variation in size of bTB breakdowns in Northern Ireland.

Model	AIC
M4: herd size+contact tracing+genotype	2737
M3:contact tracing+genotype	2827
M2:herd size+genotype	2919
M1: Null model (genotype only)	2983

Models listed in order of decreasing goodness of fit (increasing AIC).

**Table 3:** Parameter estimates from best fitting linear regression model explaining variation in the size of bTB breakdowns in Northern Ireland.

Effect	Estimate	S.E.	Z	*P*	n
Intercept (AHT)	−0.45	0.122	−3.66	<0.001	690
LRS	−0.31	0.068	−4.58	<0.001	431
LCT (lateral)	0.49	0.067	7.39	<0.001	334
BCT (backward)	1.15	0.136	8.44	<0.001	49
CTS (check)	1.67	0.330	5.05	<0.001	7
FCT (forward)	−0.04	0.323	−0.11	0.909	11
CTT (forward)	−0.14	0.112	−1.26	0.207	102
Herd size (log)	0.25	0.026	9.65	<0.001	N/A

Detection mode abbreviations: AHT = annual herd test, LRS = lesions detected at routine slaughter, LCT = lateral check test, BCT = backward check test, FCT = forward check test, CTT = check test trace. Herd size parameter represents the increase in breakdown size with increasing log herd size.

There were no significant differences in mean size of breakdowns caused by different *M. bovis* genotypes; in all of the models fitted the variance among genotype estimates was zero. Confirming these findings, there was no significant loss of fit when we compared the best fitting model (M4) with a simpler model that did not incorporate genotype level variation (Likelihood ratio test *Χ* ^2^ = 0, *d.f.* = 1, *P* = 0.999, log-likelihood identical for both models). There was considerable residual size variation among breakdowns, with a standard deviation of breakdown sizes estimated with the best fitting model of 0.8. We found no evidence of variation in mean breakdown size among spoligotypes; models separating VNTR and spoligotype effects revealed very similar patterns to those using the compound genotype classification.

### Virulence

**Figure 2:**
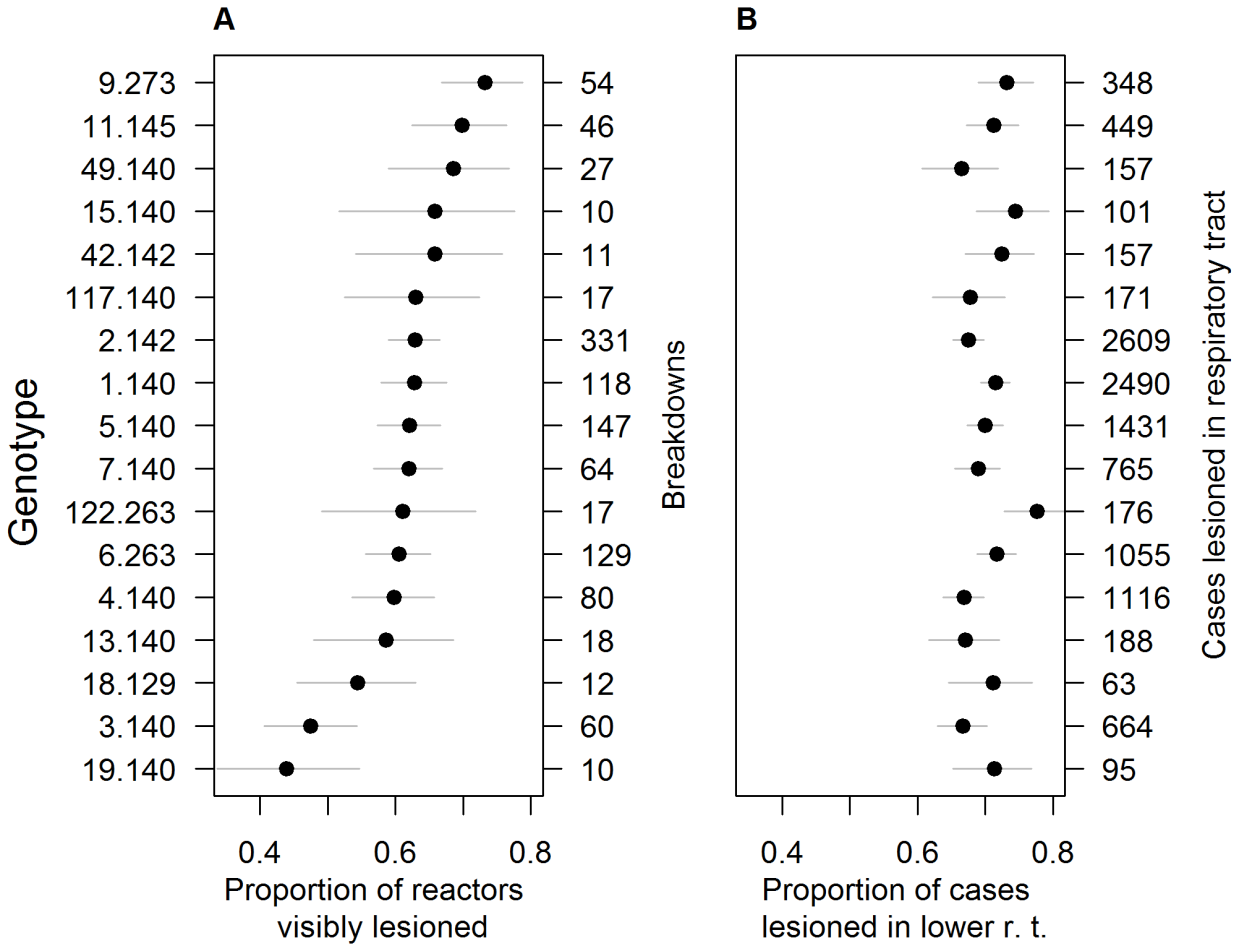
Variation among *M. bovis* genotypes in proportion of reactors visibly lesioned and distribution of lesions. Variation among *M. bovis* genotypes in A) the proportion of reactors found to be visibly lesioned (mean and 95% CIs) and B) the proportion of cases with respiratory tract lesions having lesions in the lower tract. The seventeen most abundant genotypes are plotted.

The mean proportion of reactors visibly lesioned in a breakdown varied among *M. bovis* genotypes. Estimates ranged from 44% of reactors lesioned in breakdowns caused by genotype 19.140 to 73% lesioned for genotype 9.273 (Figure 2A), and there were some significant differences among genotypes (genotypes are named [VNTR type.Spoligotype], e.g. genotype 19.140 = VNTR type 19, spoligotype SB0140). For example, animals infected with genotype 3.140 were significantly less likely to be lesioned than those infected with genotype 11.145 (95% CIs do not overlap, Figure 2A). The variance among genotypes decreased slightly (from 0.128 to 0.115) when breakdowns with only one case were excluded but the order of genotype effects remained very similar (Pearson’s *r* = 0.94, *d.f.* = 41). The proportion of reactors lesioned decreased with cattle age (regression coefficient = −0.66, *z* = −10.6, *P*<0.001) and was also influenced by the manner in which an outbreak was detected: those detected by backward or lateral tracing of infected animals had lower proportions of lesioned reactors than those detected at annual herd tests (Likelihood ratio test, contact tracing vs. non-contact tracing model: *Χ* ^2^ = 16.9, *d.f.* = 6, *P* = 0.010). Estimated genotype effects were closely correlated across both models (Pearson’s *r* = 0.99, *d.f.* = 66). Therefore it is unlikely that differences among outbreaks due to the manner of detection, or the interpretation of the skin test in large and small outbreaks were responsible for the observed inter-genotype variation in proportion of reactors lesioned. We found no systematic differences in the proportion of reactors lesioned among spoligotypes (genotypes with different spoligotypes interspersed throughout the range of responses, Figure 2A).

**Figure 3:**
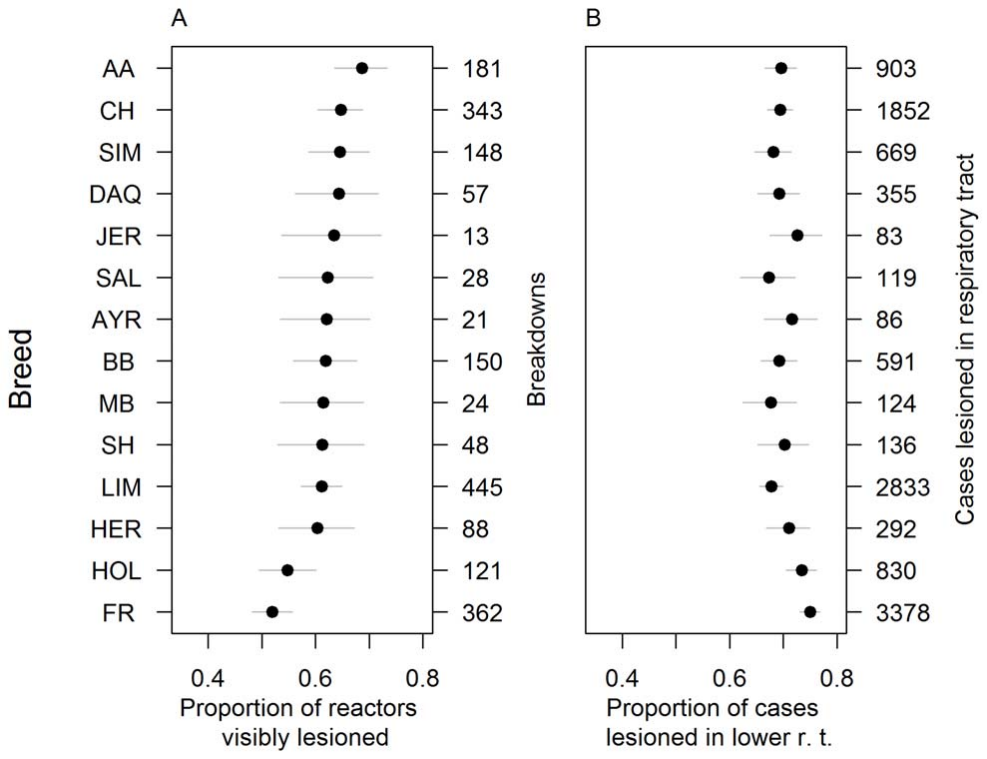
Variation among cattle breeds in proportion of reactors visibly lesioned and distribution of bTB lesions. The fourteen most abundant breeds are plotted. Breeds: AA = Aberdeen Angus, AYR = Ayrshire, BB = Belgian Blue, CH = Charolais, DAQ = Blonde D’Aquitaine, FR = Friesian, HER = Hereford, HOL = Holstein, JER = Jersey, LIM = Limousin, MB = Montbeliarde, SAL = Saler. SH = Shorthorn, SIM = Simmental.

We found less variation among cattle breeds than among *M. bovis* genotypes in the proportion of reactors lesioned (means ranged from 52% of Friesians to 69% of Aberdeen Angus), and estimates for the majority of breeds did not differ significantly from one another (overlapping CIs, Figure 3A).

### Lesion Distribution

Lesion sites varied according to cattle age and pathogen genotype and to a lesser extent with cattle breed. The majority of lesions were found in the respiratory tract indicating that this is the most common route of infection of cattle in Northern Ireland. Only 1.8% of animals were found to have lesions associated with the digestive tract (i.e. the mesenteric lymph nodes). Overall 70% of lesions in the respiratory tract were found in the lower section (i.e. lungs or bronchio-mediastinal lymph nodes), and this proportion increased significantly with animal age. The mean age of animals at slaughter in our dataset was 50 months. Predictions from our fitted regression model indicated that 63% of animals slaughtered at 20 months (lower age quartile) would be lesioned in the lower tract, increasing to 75% at 72 months (upper age quartile).

The proportion of respiratory tract lesions found in the lower tract varied slightly among genotypes, with estimates ranging from 66% for animals infected with genotype 49.140 to 77% for animals with genotype 122.263. Uncertainty around these estimates means that very few of the genotypes effects can be considered to be significantly different from one another, with the majority of 95% CIs overlapping (Figure 2B). In addition, genotype effects were not clustered by spoligotype. There was less variation in lesion site among cattle breeds, with estimated proportions of cases lesioned in the lower tract ranging between 67% and 75% (Figure 3B). The most pronounced difference was between Friesians, which had fewer cases lesioned in the upper tract than the major beef breeds (e.g. Aberdeen Angus, Charolais, Simmental, Limousin).

We found no association between genotype virulence and the lesion distribution. There was only a very weak correlation between genotype estimates of the proportion of reactors visibly lesioned with the proportion of lesioned cases that had lesions in the lower respiratory tract (Pearson *r* = 0.15, *d.f.* = 21, c.f. Figures 2A and 2B). There was a weak negative correlation between these two estimated proportions among cattle of different breeds (Pearson *r* = −0.60, *d.f. = *33, Figures 3A and 3B).

## Discussion

### Breakdown Size

We found no differences in the average breakdown size of herds infected with different genotypes of *M. bovis* when the effects of herd size and contact tracing had been accounted for. These results were partially consistent with the single previous study to investigate variation in transmissibility of *M. bovis* (in Great Britain) which indicated that there was no significant variation in outbreak size among VNTR types, although there was subtle variation among spoligotypes (a coarser level of classification) which we did not find [16].

Comparisons of multi-drug resistant with drug susceptible strains of *M. tuberculosis* revealed pronounced differences in transmissibility, although the effect size and direction was highly dependent on the strains compared and the design of the individual study [34]. In contrast, there was no evidence of variation in transmissibility or virulence in a comparison of field isolated *M. tuberculosis* strains that were distinguished by genotype alone rather than by clinical characteristics [35]. However in a population based survey of TB case cluster sizes in Malawi, strains identified using genetic markers were shown to vary in transmissibility. Cluster sizes were also strongly affected by mixing patterns within the host population, with sociable patient groups strongly represented in larger clusters [36].

Herd size and the means by which infection was detected (contact tracing) were related to breakdown size, highlighting the importance of host population structure in determining *M. bovis* transmission rates, as expected based on field studies and mathematical models of *M. tuberculosis* transmission [37]–[39]. Simulations of human TB transmission indicate that variation in susceptibility within host populations can result in considerable variation in outbreak sizes even when pathogen strains are assumed to be equally transmissible [40]. Multiple risk factors, both genetic and husbandry related have been identified which may modulate cattle susceptibility to bTB at the animal level [9]. Outbreak size can also be affected by the presence of other diseases in the population; patients infected with HIV are at greater risk of TB and an HIV epidemic can significantly increase the size of subsequent TB outbreaks [41]. In cattle herds there is some evidence that liver fluke (*Fasciola hepatica*) can influence host susceptibility and also modify the sensitivity of the skin test, potentially allowing large breakdowns to develop undetected [42]. In some situations herd management may affect breakdown size; *M. bovis* transmission within intensively managed dairy herds in Spain was shown to be faster than that within herds managed for beef or to provide animals for bullfighting, although once detected, breakdowns could be more easily controlled in dairy herds [37].

Having accounted for herd size and contact tracing we still found considerable residual variation in breakdown sizes with a small number of breakdowns much larger than the average. This feature of the breakdown size distribution may be related to variation among herds in the time since infection was introduced but could also be the result of superspreading, whereby a small number of hosts are responsible for a large number of secondary infections and have a large influence on epidemic progression [43], [44]. Superspreading may occur when there is contact between an infected individual and a large number of susceptible individuals over a short time period [45], for instance when cattle are mustered for TB testing or for milking.

A similar effect may occur when a few individuals are responsible for shedding a disproportionately large number of infectious particles into the environment (supershedding). For example, a small number of patients in a hospital ward who received inadequate treatment for multi-drug resistant TB were responsible for 90% of transmission to a sentinel animal host [46]. Pathogen strain may also influence supershedding; particular strains of *E. coli* have been shown to induce supershedding in cattle, leading to many more secondary cases than other strains [47]. Shedding in cattle infected with *M. bovis* can be intermittent, with estimates indicating that only 9–19% of animals shed in nasal or tracheal secretions [48], which might explain some of the observed variation in outbreak sizes. Variation among animals in infectious dose received (potentially as a result of supershedding) may also have influenced our measures of virulence and pathology. Cattle experimentally infected with *M. bovis* showed patterns of variation in the size and distribution of lesions that were associated with infectious dose [49]. However infectious doses in natural infection events are likely to be less variable than within these studies (where challenge doses ranged up to 1×10^7^ colony forming units in some cases [50]) and so we would not expect this effect to be pronounced in our dataset.

### Virulence

Breakdowns associated with some pathogen genotypes had a greater proportion of lesioned reactors than others, indicating that there are subtle differences in the virulence of genotypes, an effect observed in one previous field study of *M. bovis* [16]. Variation in virulence among strains of *M. tuberculosis* has been found in experimental settings on multiple occasions [51], [52]. In one such study, strains that were commonly found in large clusters of cases were compared with those that found singly. Clustered strains were found to have a more virulent phenotype, invading human macrophages *in vitro* more rapidly than non-clustered strains and inducing different cytokine responses, giving clues as to the mechanism of invasion [15]. *M. bovis* strains have also been shown to vary in virulence, eliciting strain specific patterns of immune response in mice [53]. A potential line for future investigation would be to compare the genotypes which we identified as differing significantly in virulence (e.g. 9.273 and 19.140), perhaps using transcriptomic techniques to elucidate the mechanisms driving the observed variation [54]. Alternatively, the genes responsible might be identified by mapping virulence traits onto a phylogeny of genotypes. Given the close relationships among genotypes in Northern Ireland, whole genome sequencing of *M. bovis* isolates might be required to construct such a phylogeny [55].

A potential drawback to our choice of virulence measure (i.e. the proportion of reactors lesioned) is that it might have been influenced by variation in detectability of genotypes to the skin test. If certain genotypes were less detectable to the skin test then recently infected animals (probably not lesioned) might remain undetected, decreasing the total number of reactors and thus increasing the observed proportion lesioned. However we consider this possibility unlikely because in a parallel study we found no systematic differences among genotypes using two different measures of skin test detectability [56].

Laboratory studies indicate that more virulent strains of *M. tuberculosis* are also more transmissible than less virulent strains and tend to form larger outbreaks within human populations [57], [58]. In contrast, we found evidence of variation among *M. bovis* genotypes in virulence but not in transmissibility. A possible explanation is that the current test and slaughter programme is sufficient to prevent differences in virulence being manifested in terms of outbreak size, with even the most virulent strains being detected prior to large-scale onward spread. Indeed, the majority of cases show limited pathology (1–2 lesions detected) indicating relatively recent infection and cases of generalised bTB (systemic infection with lesions in organs not connected to the respiratory or alimentary tract [50]) are rare in Northern Ireland.

### Lesion Distribution

We found variation in pathology induced by different genotypes of *M. bovis*, with subtle differences in lesion location. A study of *M. tuberculosis* infection in humans linked differences in pathology to strain virulence by comparing isolates taken from a group of patients simultaneously infected with two strains, one disseminated and the other localised. Disseminated strains were found to have greater virulence in laboratory assays than localised strains [59]. We found no such relationship (no correlation between proportion of reactors lesioned and proportion of cases lesioned in lower respiratory tract) perhaps because our measure of lesion distribution was derived from cases that were in relatively early stages of infection where only a single lesion was found. The variation in lesion distribution that we observed was therefore more likely to have been linked to processes determining establishment of the initial infection rather than factors governing disease progression, and the former warrants further investigation.

The observed differences in infection site among cattle breeds may be related to variation in animal husbandry. Lesion sites differed in a comparison of groups of cattle kept indoors in Ethiopia with those kept outdoors; animals under intensive management indoors were more likely to be lesioned in the lower respiratory tract, indicating the respiratory route of infection, than those kept outdoors. Animals kept indoors were also more likely to have more severe pathology and an increased risk of acquiring infection [20], [60]. We found a similar pattern with Friesian and Holstein cattle (primarily dairy breeds) in particular showing evidence of a greater degree of infection through the respiratory route (a higher proportion of cases lesioned in the lower respiratory tract) than most of the beef breeds, although the differences that we observed among breeds were relatively small. We also found differences between Holstein-Friesian cattle and beef breeds in the proportion of reactors lesioned, with a greater proportion of reactors belonging to beef breeds having visible lesions. A potentially informative area of future research would be to use measures of pathology to investigate links between husbandry (including stocking density) and the risk of infection by the different routes.

Besides differences in husbandry, the observed variation in lesion distribution may also indicate genetic differences in TB susceptibility among breeds. European *Bos taurus* cattle breeds in Ethiopia have been shown to be more susceptible than native *Bos indicus* cattle [61]. There is also evidence of heritable variation in TB susceptibility within breeds in Irish cattle [62] and so our findings are consistent with the view that host genetic variation influences the outcome of exposure to *M. bovis* and that knowledge of this variation may have a role in future disease control programmes [63]–[65].

The limited genetic diversity of the *M. bovis* population in Northern Ireland may explain the relatively subtle differences that we found in virulence and lesion distribution. Genotypes of *M. bovis* in the UK and Ireland belong almost exclusively to the EU1 clonal complex which has much less diversity at the spoligotype level than the population in continental Europe, where EU1 is relatively rare [66]. Reduced diversity in Great Britain has been attributed to a series of population bottlenecks, the most recent being the introduction of a comprehensive ‘test and slaughter’ control programme [67]. Diversity is further restricted in Northern Ireland, where 96% of isolates belong to the dominant spoligotype (SB0140) or its derivations [13].

### Conclusions

Using a combination of genotyping and epidemiological data we investigated the associations between *M. bovis* genotypes and patterns of outbreak sizes, virulence and pathology in naturally occurring cases across Northern Ireland during a nine year period. Some genotypes were associated with a greater proportion of lesioned cases indicating that genotypes differ in virulence. However, we found no evidence for systematic variation in breakdown sizes among genotypes, perhaps indicating that the programme of annual skin testing and abattoir surveillance is successfully preventing more virulent and transmissible genotypes from causing large outbreaks. Cases infected with different genotypes also varied in the distribution of lesions and there was variation in lesion distribution among cattle breeds, perhaps indicative of different disease susceptibility and transmission routes in beef and dairy cattle, traits which with further investigation might be exploited to aid disease control.
